# Characterization of autism spectrum disorder and neurodevelopmental profiles in youth with XYY syndrome

**DOI:** 10.1186/s11689-018-9248-7

**Published:** 2018-10-22

**Authors:** Lisa Joseph, Cristan Farmer, Colby Chlebowski, Laura Henry, Ari Fish, Catherine Mankiw, Anastasia Xenophontos, Liv Clasen, Bethany Sauls, Jakob Seidlitz, Jonathan Blumenthal, Erin Torres, Audrey Thurm, Armin Raznahan

**Affiliations:** 1 Office of the Clinical Director, National Institute of Mental Health, National Institute of Health, Bethesda, MD USA; 2 Developmental Neurogenomics Unit, Human Genetics Branch, National Institute of Mental Health, National Institutes of Health, Bethesda, MD USA

**Keywords:** Sex chromosome aneuploidies, Autism spectrum disorder symptoms, Learning disabilities, Adaptive behavior, Cognitive functioning

## Abstract

**Background:**

XYY syndrome is a sex chromosome aneuploidy that occurs in ~ 1/850 male births and is associated with increased risk for neurodevelopmental difficulties. However, the profile of neurodevelopmental impairments, including symptoms of autism spectrum disorder (ASD) in XYY remains poorly understood. This gap in knowledge has persisted in part due to lack of access to patient cohorts with dense and homogeneous phenotypic data.

**Methods:**

We evaluated a single-center cohort of 64 individuals with XYY aged 5–25 years, using a standardized battery of cognitive and behavioral assessments spanning developmental milestones, IQ, adaptive behavior, academic achievement, behavioral problems, and gold-standard diagnostic instruments for ASD. Our goals were to (i) detail the neurodevelopmental profile of XYY with a focus on ASD diagnostic rates and symptom profiles, (ii) screen phenotypes for potential ascertainment bias effects by contrasting pre- vs. postnatally diagnosed XYY subgroups, and (iii) define major modules of phenotypic variation using graph-theoretical analysis.

**Results:**

Although there was marked inter-individual variability, the average profile was characterized by some degree of developmental delay, and decreased IQ and adaptive behavior. Impairments were most pronounced for language and socio-communicative functioning. The rate of ASD was 14%, and these individuals exhibited autism symptom profiles resembling those observed in ASD without XYY. Most neurodevelopmental dimensions showed milder impairment among pre- vs. postnatally diagnosed individuals, with clinically meaningful differences in verbal IQ. Feature network analysis revealed three reliably separable modules comprising (i) cognition and academic achievement, (ii) broad domain psychopathology and adaptive behavior, and (iii) ASD-related features.

**Conclusions:**

By adding granularity to our understanding of neurodevelopmental difficulties in XYY, these findings assist targeted clinical assessment of newly identified cases, motivate greater provision of specialized multidisciplinary support, and inform future efforts to integrate behavioral phenotypes in XYY with neurobiology.

**Trial registrations:**

ClinicalTrials.gov NCT00001246, “89-M-0006: Brain Imaging of Childhood Onset Psychiatric Disorders, Endocrine Disorders and Healthy Controls.”

## Background

Sex chromosome aneuploidy is the carriage of sex chromosome complements other than XX in females or XY in males, and is estimated to occur in 1/420 live births [1]. One of the most common is XYY syndrome, with an estimated prevalence of ~ 1/850 male births [1]. Longstanding interest and controversy regarding the behavioral phenotype of XYY was sparked by an early series of influential yet methodologically flawed case-control studies suggesting an association between XYY and commission of violent crimes [2]. More rigorous research in larger cohorts has robustly refuted this association [3, 4], further noting that individuals with XYY may be at increased risk for a range of neurodevelopmental difficulties [4–8].

To date, the neurodevelopmental phenotype of XYY has been most consistently associated with lowered intelligence quotient (IQ), language impairments, academic problems, and difficulties with attention and social interaction [9]. Studies of general cognitive ability in XYY report IQ deficits of approximately 10 points [4, 10], preferentially affecting verbal domains [4]. Relatedly, language delay [10, 11] and subsequent language impairments are consistently reported in both birth and clinical cohorts [4, 6]. The increased rates of academic difficulties and special education needs [4, 6, 12, 13] are most pronounced in the reading domain [12, 13]. XYY has also been associated with impairments in adaptive functioning, potentially exceeding that predicted by lowered IQ alone [7]. Consistent with the documented reductions in IQ and adaptive functioning, rates of intellectual disability are increased relative to the general population [8], as are other neurodevelopmental disorders including attention deficit hyperactivity disorder [8] and autism spectrum disorder (ASD) [7, 14].

The potential association between XYY and ASD has undergone intense study [6, 7, 14–16], motivated in part by an unbalanced sex ratio of ASD among karyotypically normal children who are diagnosed with ASD [17]. The most methodologically rigorous study yielded an ASD diagnostic rate of 38% in 57 participants [7]. However, it is not known whether the phenotypic presentation of ASD in youth with XYY is distinguishable from those with idiopathic ASD, and whether ASD-related features reliably segregate with other phenotypic aspects of XYY.

Here, we examine the neurodevelopmental phenotype of XYY in depth within a previously unpublished cohort of 64 youth aged 5–25 years, representing the largest single-center study of XYY. Our study was designed to build upon existing knowledge regarding neurodevelopment in XYY in three key directions.

First, there are no existing studies that contemporaneously capture the full range of neurodevelopmental dimensions using homogenous single-center protocols with a common set of instruments across all participants. We sought to achieve dense and homogenous phenotypic characterization within a large cohort to detail multiple developmental dimensions in XYY, and systematically examine inter-relationships between different aspects of the neurodevelopmental profile.

Second, although elevated rates of ASD in XYY relative to the general population are noted in independent reports [6, 7], studies have not uniformly applied a gold-standard ASD diagnostic battery for all participants. Thus, we address this need by focusing on updating the estimate of the ASD diagnostic rate in groups identified with XYY, qualitatively comparing the profile of ASD features in XYY to ASD without XYY and capturing relationships between ASD and other developmental phenotypes. To meet these goals, we gathered a diverse set of ASD-related measures that can support research-level diagnostic assessment (i.e., Autism Diagnostic Observation Schedule, second edition (ADOS-2; [18]), Autism Diagnostic Interview, Revised (ADI-R; [19]) and provide complementary dimensional measures of ASD-related traits (i.e., Social Responsiveness Scale, second edition (SRS-2; [20]), Social Communication Questionnaire (SCQ; [21]) and Repetitive Behavior Scale, Revised (RBS-R; [22]). Finally, the potential for ascertainment bias has continued to pose a major challenge for studies of neurodevelopment in XYY, given the likely low detection rate and the fact that neurodevelopmental difficulties often precipitate testing in postnatally diagnosed cases [23]. Here, we harness differences between pre- and postnatally identified XYY subgroups [5, 15] as a proxy test for potential ascertainment bias effects.

## Methods

### Participants

Singleton males (*N* = 64) aged 5 to 25 years with XYY were enrolled in a phenotypic characterization study at the National Institutes of Health (NIH) (Table 1). Informed consent and assent was obtained from all participants and their parents; all study procedures were approved by an NIH Institutional Review Board. Participants were recruited through the Association for X and Y Chromosome Variations (AXYS; genetic.org) and the NIH Clinical Center Office of Patient Recruitment. XYY was confirmed and mosaicism ruled out by karyotype testing of a minimum of 50 metaphases, either through the study or confirmed from community karyotype reports when blood draw was not possible.

**Table 1:** Participant characteristics by time of diagnosis

	Full sample (*N* = 64)	Prenatal/birth diagnosis (*n* = 25)	Later diagnosis (*n* = 39)	Prenatal/birth: later comparison
	*n (%)*, M ± SD, or median [IQR]			
Demographic information				
Age (years)	13.10 ± 5.72	13.66 ± 6.27	12.74 ± 5.39	*t*(62) = 0.62, *p* = .53
Socioeconomic status	52.78 ± 18.73	47.52 ± 15.91	56.24 ± 19.82	*Z* = 1.31, *p* = .19
Prior (community) ASD diagnosis	22 (34%)	7 (28%)	15 (38%)	*χ*^*2*^ = 0.74, *p* = .39
Current DSM-5 ASD diagnosis	9 (14%)	2 (8%)	7 (18%)	Fisher’s exact *p* = .30
Early intervention (current or history)	22 (34%)	10 (40%)	12 (31%)	*χ*^*2*^ = 0.58, *p* = .45
Speech therapy (current or history)	44 (69%)	14 (56%)	30 (77%)	*χ*^*2*^ = 3.10, *p* = .08
Special education (current or history)	55 (86%)	19 (76%)	36 (92%)	Fisher’s exact *p* = .14
Any intervention (current or history)	59 (92%)	21 (84%)	38 (97%)	Fisher’s exact *p* = .07
Developmental Milestones				
Age of first words (months)	24 [18–36]	19 [18–24]	24 [18–36]	*Z* = −1.19, *p* = .24
Age of phrases (months)	36 [26–42]	26 [24–30]	42 [30–48]	*Z* = −2.94, *p* = .003
Age walked independently (months)	14 [13–18]	15 [12–18]	14 [13–18]	*Z* = −0.35, *p* = .73
Daytime toilet training (months)	37.5 [31–45]	36 [32–48]	39 [30–42]	*Z* = 0.02, *p* = .99
Nighttime toilet training (months)	42 [36–72]	42 [36–84]	42 [36–60]	*Z* = 0.53, *p* = .60
Bowel control toilet training (months)	37.5 [31–45]	36 [32–42]	39 [30–48]	*Z* = −0.71, *p* = .48
Cognitive ability				
Visual-spatial	97 [89–106]	100 [92–112]	94 [86–100]	*Z* = 1.92, *p* = .05
Fluid reasoning	93 ± 14.46	97.06 ± 12.88	90.7 ± 14.99	*t*(45) = 1.47, *p* = .15
Working memory	88.92 ± 14.53	91.04 ± 13.64	87.5 ± 15.12	*t*(58) = 0.92, *p* = .36
Processing speed	83.67 ± 14.17	89.75 ± 14.02	79.61 ± 12.92	*t*(58) = 2.88, *p* = .01
NVIQ	91.81 ± 15.56	95.76 ± 14.24	89.28 ± 16.01	*t*(62) = 1.65, *p* = .10
VIQ	85.89 ± 14.18	91.56 ± 13.97	82.16 ± 13.2	*t*(61) = 2.7, *p* = .01
FSIQ	86.24 ± 13.49	90.4 ± 12.17	83.5 ± 13.77	*t*(61) = 2.04, *p* = .05
Academic achievement				
Woodcock-Johnson Math	85 [77–99]	93 [77–100]	80.5 [75–93]	*Z* = 1.24, *p* = .21
Math disability	25/53 (47%)	6/18 (33%)	19/35 (54%)	*χ*^*2*^ = 2.09, *p* = .15
Woodcock-Johnson Writing	93 [73–99]	95 [92–101]	92 [73–97]	*Z* = 1.24, *p* = .21
Writing disability	19/50 (38%)	6/18 (33%)	13/32 (41%)	*χ*^*2*^ = 0.26, *p* = .61
Woodcock-Johnson Reading	85 [70–93]	88 [74–95]	83.5 [67.5–91]	*Z* = 0.99, *p* = .32
Reading disability	25/52 (48%)	9/19 (47%)	16/33 (48%)	*χ*^*2*^ = 0, *p* = .94
Adaptive behavior				
Vineland communication	77.69 ± 15.04	81.8 ± 17.06	75.05 ± 13.15	*t*(62) = 1.78, *p* = .08
Vineland daily living skills	79.11 ± 14.55	82.12 ± 14.66	77.18 ± 14.33	*t*(62) = 1.33, *p* = .19
Vineland socialization	77.2 ± 14.01	81.64 ± 14.35	74.36 ± 13.19	*t*(62) = 2.08, *p* = .04
Vineland ABC	76.22 ± 12.95	79.68 ± 13.65	74 ± 12.14	*t*(62) = 1.74, *p* = .09
Autism symptoms				
SRS total *T* score	66.21 ± 7.86	62.08 ± 7.82	68.82 ± 6.76	*t*(60) = − 3.59, *p* = .001
ADOS social affect CSS	2 [1–5]	2 [1–4]	2 [1–5]	*Z* = − 0.6, *p* = .55
ADOS RRB CSS	1 [1–6]	1 [1–5]	5 [1–7]	*Z* = − 2.05, *p* = .04
ADOS CSS	2 [1–3]	1 [1–3]	2 [1–3]	Z = − 1.05, *p* = .29
RBS-R overall total	13 [6–27]	7 [3–14]	19 [9–31]	*Z* = −2.72, *p* = .01
Other psychopathology				
CBCL internalizing behavior	61.40 ± 8.29	58.00 ± 7.58	63.55 ± 8.08	*t*(60) = −2.7, *p* = .01
CBCL externalizing behavior	59.39 ± 12.31	56.71 ± 11.27	61.08 ± 12.77	*t*(60) = −1.37, *p* = .18

Variables that were not normally distributed (significant Shapiro-Wilk statistic) are described with median and interquartile range and tested using the Wilcoxon rank-sum test. Normally distributed variables are described with mean and standard deviation and tested using an independent samples *t* test (where DF have decimals, Satterthwaite approximation was used)

### Measures

#### Developmental history

Timing of developmental milestones (i.e., first words, first use of phrases, independent walking, continence) was obtained using the ADI-R [19], which also queried intervention services and existing neurodevelopmental diagnoses. The timing of XYY diagnosis (either prenatal/birth or postnatal) was based on parent report.

#### Socioeconomic status

The Hollingshead two-factor index was used to assess socioeconomic status of participants, with education and occupation factors included.

#### Cognitive ability

The Wechsler Preschool and Primary Scale of Intelligence, fourth edition, Wechsler Intelligence Scale for Children, fifth edition, or Wechsler Adult Intelligence Scale, fourth edition was used to assess intelligence. If the participant had been tested with a Wechsler scale within 1 year (*n* = 4), the Wechsler Abbreviated Scale of Intelligence, second edition was used.

#### Adaptive function

The Vineland Adaptive Behavior Scales, second edition (VABS; [24]) is a standardized semi-structured caregiver interview to assess adaptive function in the domains of communication, daily living skills, socialization, and motor skills.

#### Academic achievement

The Woodcock-Johnson Tests of Achievement, fourth edition (WJ-IV) is a psychoeducational assessment of academic achievement.

#### Neurodevelopmental and behavioral phenotyping

The ASD diagnostic battery had three components: the ADOS-2, the ADI-R, and consensus of at least two clinicians in completing the DSM-5 diagnostic criteria checklist [25]. Assessments were performed by licensed clinical psychologists (L.J., A.T., C.C.) with extensive ASD evaluation experience, who met research reliability standards on the ADI-R and ADOS-2.

Caregiver-rated screening questionnaires were also used to assess ASD-related symptoms. These included the SRS-2, the SCQ, and the RBS-R. Other behavioral problems were assessed using either the Child Behavior Checklist or the Adult Behavior Checklist, depending on the age of the participant (referred to collectively as CBCL).

### Statistical analyses

Variables were assessed for normality prior to analysis; group differences for those with significant Shapiro-Wilk statistics were analyzed with a nonparametric alternative (Wilcoxon rank sum). Normally, distributed variables were assessed using *t* tests (independent samples for comparisons between participants diagnosed prenatally vs. at birth, or single-sample for comparison to population norms), with Satterthwaite-adjusted values in the presence of unequal variance.

To facilitate graphical comparison of the XYY sample to the ASD population, scores were *Z* normalized against ASD normative data drawn from the Simons Simplex Collection (SSC), a research cohort ascertained primarily from autism clinics with gold-standard diagnostic measures used to confirm diagnoses; we restricted this sample to include only males (*N* = 1877). For consistency with the SSC ASD data, the XYY sample was restricted to include participants younger than 18 years (*n* = 48).

Psychometric properties were quantified for the ASD scales. Sensitivity and specificity, as well as the area under the curve (AUC) were calculated against DSM-5 ASD diagnosis. These values were also calculated for participants with and without behavior problems, as indicated by CBCL internalizing or externalizing *T* scores greater than or equal to 64.

The inter-relationships among phenotypic variables in XYY were examined as follows. Any variables with a scaled mean correlation with other variables of less than − 2 were removed (SRS-2 awareness score, and age at walking independently). Matrices of pairwise Pearson correlations for the remaining variables were generated, using 1000 separate bootstrap draws of 64 individuals (with replacement). Each matrix was submitted to hierarchical clustering, using the gap statistic method [26]. A single square adjacency matrix was constructed using the proportion of times variable pairs were co-clustered across all 1000 analyses. Finally, a network representation of this adjacency matrix was used to define modules of phenotypic variables based on the consensus of 1000 runs of the Louvain algorithm in the MATLAB Brain Connectivity Toolbox. The Louvain algorithm gamma value was set at 1.2 by defining the local minimum of the global mean nodal versatility curve [27].

Alpha was set to .05 to reflect the descriptive nature of this report. Analyses were performed in SAS/STAT Version 9.3 and R 3.3.0 [28]; graphics were created using the igraph [27], superheat [29], and ggridges [30] packages in R 3.3.0.

## Results

The sample ranged in age from 5 to 25 years and was predominantly white (*n* = 58, 91%). The majority (*n* = 39, 61%) received their XYY diagnosis sometime after birth (mean age of diagnosis = 6.08 ± 4.55, range 0.02 years to 16.48 years).

**Fig. 1:**
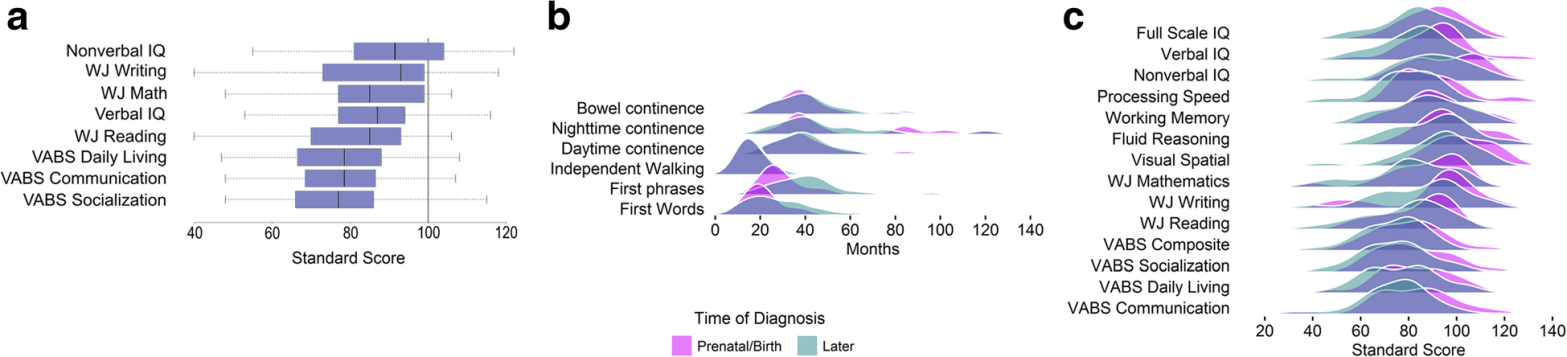
Phenotypic profiles. **a** All differences from the population mean of 100 are statistically significant, *p* < .0001. **b** Milestones. Two participants with extreme continence data (> 140 months) are not represented. **c** Neurodevelopmental phenotype

Table 1 and Fig. 1 summarize ratings across all neurodevelopmental domains examined in our cohort. Impairments were greatest for language and socio-communicative functioning, and least for math and non-verbal domains (Fig. 1a). Individual domains are considered separately below.

### Early development and intervention history

Using a threshold of 24 months [31], we observed delays in the median age of single word acquisition, phrase speech, and independent walking. Median ages for continence milestones were in the fourth and fifth years, constituting delay [32]. Those who were diagnosed prenatally did not differ from those diagnosed later in age of first words, but their median age of phrase speech was about 16 months earlier (see Table 1; Fig. 1b). The groups did not differ on other milestones, walking, daytime continence, nighttime continence, and bowel continence. By parent report, nearly all participants had received some sort of intervention during childhood (see Table 1), though only about one-third received some type of intervention service prior to the age of 3 years.

### Cognitive profile

One participant, whose first language was not English, received only the non-verbal battery. Among the remaining 63 participants, full-scale IQs (FSIQ) ranged from 53 to 112 (*n* = 34, 54% in the average range, *n* = 22, 35% in the borderline range, *n* = 6, 9% in the mild range, and *n* = 1, 2% in the moderate range). Six participants met DSM-5 criteria for intellectual disability. The average scores for nonverbal IQ (NVIQ) and verbal IQ (VIQ) were in the low-average range, both significantly different from the population mean (non-verbal: *t*(63) = 4.21, *p* = <.0001; verbal: *t*(62) = 7.90, *p* < .0001) (see Fig. 1a). The non-verbal/verbal split was statistically significant (5.79 ± 12.75, *t*(62) = 3.61, *p* = .0006). Mean cognitive scores for participants diagnosed prenatally/at birth were higher than for those diagnosed later; differences were statistically significant only for the processing speed subscale and VIQ (Table 1).

### Academic achievement

Mean Woodcock-Johnson scores were significantly lower than the population average: Reading, (*t*(49) = − 8.66, *p* < .0001), mathematics (*t*(48) = − 6.77, *p* < .0001), and writing (*t*(45) = − 4.54, *p* < .0001) (see Fig. 1c). Per the DSM-5, criteria of at least one standard score below 78 (1.5 SD below population mean), and an FSIQ above 70 are used to indicate a specific learning disability (SLD); 63% (*n* = 37) met for at least one SLD. There was no significant difference in the rate of any SLD between participants diagnosed prenatally/at birth or postnatally (*χ*^*2*^(1) = 0.013, *p* = .91), nor were there differences in Woodcock-Johnson subscale scores (see Table 1).

### Adaptive behavior

The VABS-II Adaptive Behavior Composite scores ranged from 42 to 112, and were significantly lower than the population average (*t*(63) = 14.69, *p* < .0001). The adaptive behavior profile of the sample was relatively flat (see Fig. 1c). Socialization scores in participants diagnosed with XYY after birth were significantly lower than in participants diagnosed prenatally/at birth; communication and daily living skills did not differ between groups (see Table 1). The Adaptive Behavior Composite was significantly lower than FSIQ (*t*(62) = 6.31, *p* < .0001), and older participants tended to have more impaired Adaptive Behavior Composite scores (*r* = − 0.55, *p* = .01).

### Autism spectrum disorder

#### Diagnostic rates

Twenty-two (33%) participants carried a community diagnosis of ASD. Among the 63 participants who received ASD diagnostic measures in this study, nine (14%) participants also met criteria for a DSM-5 diagnosis of ASD. Participants with and without DSM-5 ASD did not differ in age (*t*(61) = − 0.45, *p* = .66) or FSIQ (*t*(60) = 0.47, *p* = .64), although Adaptive Behavior Composite scores were lower in those with ASD (67.22 ± 7.90 vs. 77.69 ± 13.19; *t*(61) = 2.30, *p* = .03).

#### Dimensional ratings

The median ADOS calibrated severity score was in the unaffected range (see Table 1). SRS-2 total *T* scores in this sample ranged from 50 to 89, and the mean cohort *T* score was significantly higher than the population mean of 50 (*t*(61) = 16.24, *p* < .0001).

#### Timing of XYY diagnosis and ASD

Although the rate of DSM-5 ASD diagnosis did not differ by timing of XYY diagnosis (Table 1), participants diagnosed postnatally had significantly higher SRS-2 scores, ADOS-2 Restricted/Repetitive Behavior severity scores and RBS-R Total Scores (see Table 1).

#### Sensitivity and specificity of ASD measures

The sensitivity and specificity of the diagnostic instruments (ADOS-2 and ADI-R) were high (Table 2). These psychometric profiles of the screening instruments (SRS-2 and SCQ) were more variable; the SRS-2 demonstrated excellent sensitivity and poor specificity, while the SCQ had moderate levels of both.

**Table 2:** Sensitivity and specificity of ASD measures

Stratification	ASD (*n*)	Non-ASD (*n*)	AUC (95% CI)	Sensitivity (95% CI)	Specificity (95% CI)
ADI-R					
Full sample	9	54	0.97 (0.93–1)	1.00 (0.66–1.00)	0.87 (0.75–0.95)
CBCL externalizing					
< 64	6	33	0.98 (0.96–1)	1.00 (0.54–1.00)	0.91 (0.76–0.98)
≥ 64	3	19	0.95 (0.84–1)	1.00 (0.29–1.00)	0.84 (0.60–0.97)
CBCL internalizing					
< 64	4	32	1 (1–1)	1.00 (0.40–1.00)	0.91 (0.75–0.98)
≥ 64	5	20	0.93 (0.82–1)	1.00 (0.48–1.00)	0.85 (0.62–0.97)
ADOS-2					
Full sample	9	53	0.97 (0.93–1)	0.89 (0.52–1.00)	0.98 (0.90–1.00)
CBCL externalizing					
< 64	6	32	0.99 (0.96–1)	1.00 (0.54–1.00)	1.00 (0.89–1.00)
≥ 64	3	19	0.92 (0.79–1)	0.67 (0.09–0.99)	0.90 (0.74–1.00)
CBCL internalizing					
< 64	4	31	1 (1–1)	1.00 (0.40–1.00)	1.00 (0.89–1.00)
≥ 64	5	20	0.91 (0.79–1)	0.80 (0.28–0.99)	0.91 (0.75–1.00)
SRS-2					
Full sample	9	52	0.85 (0.75–0.96)	1.00 (0.66–1.00)	0.59 (0.45–0.73)
CBCL externalizing					
< 64	6	33	0.92 (0.82–1)	1.00 (0.54–1.00)	0.69 (0.51–0.84)
≥ 64	3	19	0.72 (0.51–0.93)	1.00 (0.29–1.00)	0.42 (0.21–0.67)
CBCL internalizing					
< 64	4	32	0.96 (0.89–1)	1.00 (0.40–1.00)	0.75 (0.57–0.89)
≥ 64	5	20	0.68 (0.44–0.91)	1.00 (0.48–1.00)	0.36 (0.15–0.59)
SCQ					
Full sample	9	52	0.85 (0.73–0.96)	0.78 (0.40–0.97)	0.73 (0.61–0.85)
CBCL externalizing					
< 64	6	33	0.86 (0.72–1.00)	0.66 (0.22–0.95)	0.76 (0.58–0.89)
≥ 64	3	19	0.80 (0.60–1.00)	1.00 (0.29–1.00)	0.68 (0.43–0.87)
CBCL internalizing					
< 64	4	32	0.90 (0.80–1.00)	1.00 (0.40–1.00)	0.81 (0.64–0.93)
≥ 64	5	20	0.77 (0.56–0.98)	0.60 (0.15–0.95)	0.60 (0.36–0.81)

*ASD and non-ASD* DSM-5 diagnosis. *AUC* (area under the curve), sensitivity, and specificity refer to the comparison of the given test cut-off to the DSM-5 diagnosis

While both the sensitivity and specificity of the ADI-R were robust to the influence of additional psychopathology (measured by CBCL internalizing and externalizing), the sensitivity of the ADOS-2 was affected by high levels of externalizing behaviors (Table 2). In contrast, the specificity of the screening measures (SRS-2 and SCQ) was particularly low among participants who had clinically significant levels of internalizing or externalizing symptoms.

#### Descriptive comparison of XYY and youth with ASD

**Fig. 2:**
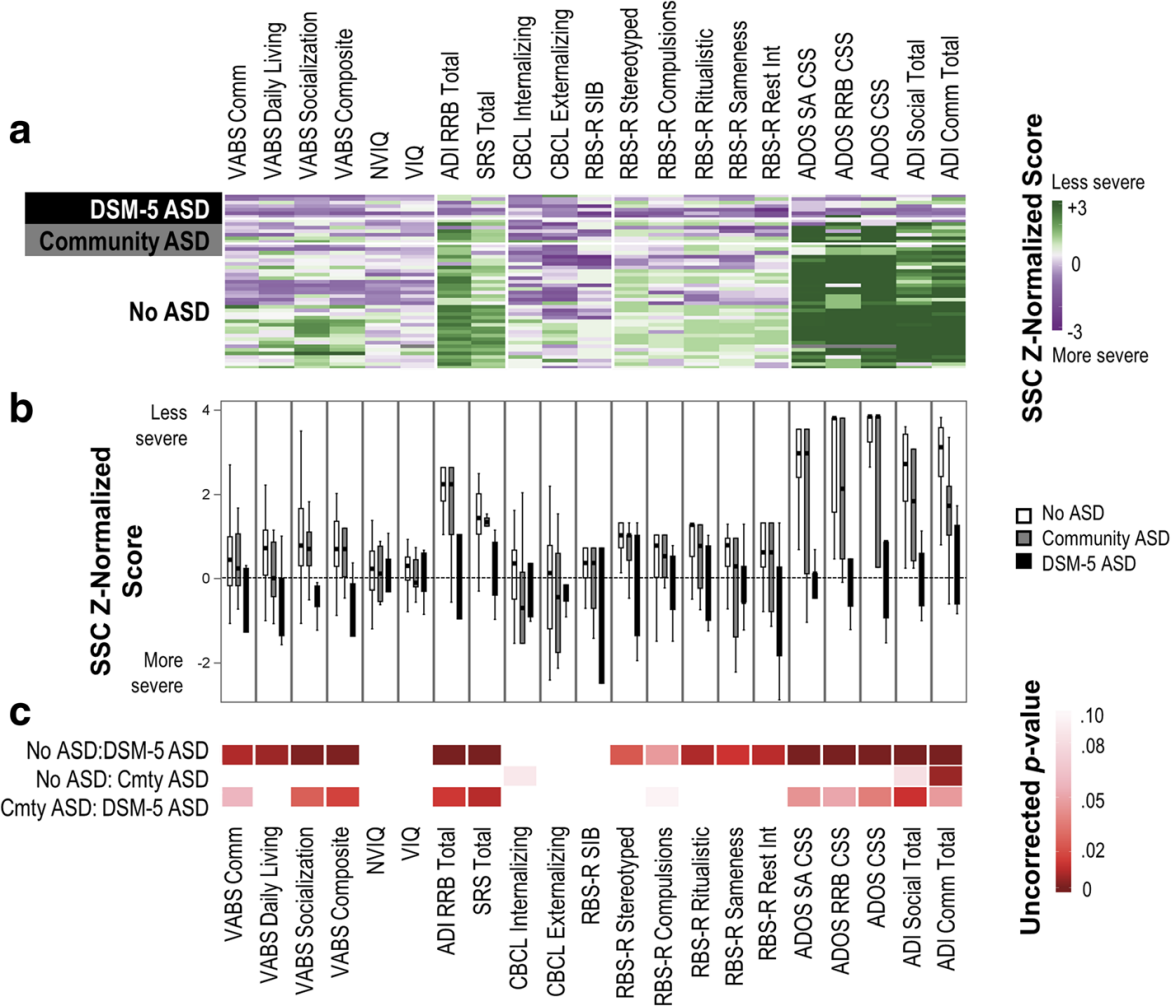
Phenotypic profiles of XYY participants younger than 18 years (*n* = 49). **a** *Z* normalized scores for each participant in the XYY sample (*Y* axis), using the means and standard deviations from the Simons Simplex Collection (SSC). **b** Boxplots of *Z* normalized scores by diagnostic status. **c** *p* values for pairwise comparisons

The scores from the XYY sample were *Z* normalized against a large sample of males with ASD (Fig. 2 a, b). Generally, the profile of children with XYY and DSM-5 ASD did not deviate from that observed in the SSC ASD sample. As expected, the profile of ASD symptoms was generally more severe among participants with DSM-5 ASD than the mean profile in subgroups without ASD or with only a community diagnosis (Fig. 2c).

### Integrative analysis of neurodevelopmental features in XYY

**Fig. 3:**
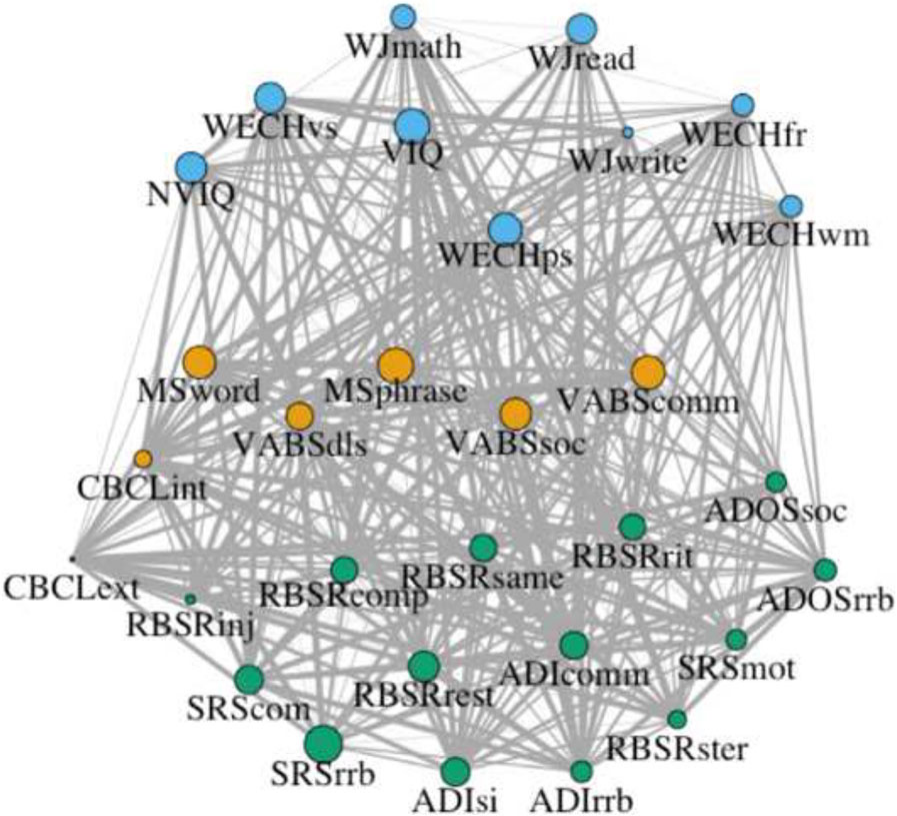
Network representation of phenotypic modules in XYY (*N* = 64). Nodes are phenotypic variables; color distinguishes reproducible clusters: cognitive ability/academic achievement (blue), adaptive functioning/psychopathology (yellow), and ASD-related features (green). Size indices the mean pairwise correlation between the variable and all others. Thicker edges show increased consistency of co-clustering based on bootstrapped analysis. The force-directed Fruchterman-Reingold Algorithm was used, such that further distance between nodes indicates weaker inter-relationships

Graph-theoretical analysis of the inter-relationship among the phenotypic variables suggested three separable sub-sets of neurodevelopmental features (Fig. 3): (i) cognition and academic achievement, (ii) broad domain psychopathology and adaptive behavior, and (iii) ASD-related features. Thus, adaptive functioning across individuals with XYY appeared to be more closely related to broad-domain psychopathology (especially internalizing symptoms vs. externalizing symptoms) than to cognitive ability. Network visualization also suggested that the cognitive and ASD-related phenotypic modules in XYY showed stronger relationships with the adaptive functioning module than they did with each other.

## Discussion

To date, the behavioral phenotype of XYY has been collectively defined using partially overlapping measures in different cohorts. The current study reinforces several key findings of these prior studies within a previously unreported single-center cohort, including delays in motor and language development (cf. [10]), wide-ranging IQ with a downward-shifted distribution and relative deficits in verbal scores (cf. [4]), and reduced adaptive functioning (cf. [8]). Our evaluations also revealed relatively low academic achievement scores, with the majority of participants meeting criteria for at least one SLD. However, we note that academic achievement scores were relatively similar between the pre- vs. postnatally diagnosed groups, despite significant group differences in a range of cognitive and behavioral domains. We speculate that this dissociation might arise due to group differences in (i) unmeasured factors (in addition to variables tested here like cognition), that contribute to academic achievement, and/or (ii) academic remediation [33, 34]. Access to dense and homogenous phenotypic data allowed us to resolve a gradient of vulnerability across different dimensions of neurodevelopment, ranging from greatest impairment in average adaptive socialization skills to least impairment in average NVIQ. These measures also helped to better-resolve potential ascertainment bias effects: we replicated the prior finding of below-average IQ among prenatally vs. postnatally diagnosed individuals with XYY (cf. [5]), and further detect statistically significant time of diagnosis effects for VIQ, processing speed, adaptive social functioning, and internalizing symptoms. This effort represents a step towards securing more accurate estimates of the penetrance of XYY for a range of neurodevelopmental issues, with the ultimate goal of informing the provision of genetic counseling in affected pregnancies.

Understanding the nature of the relationship between XYY and ASD is not only important for clinical understanding of XYY, but also for evaluating the notion that Y-chromosome dosage effects could be relevant to the male bias in ASD prevalence. While one-third of this sample had a community ASD diagnosis, only a subset of these participants met gold standard criteria for a research-based diagnosis. The rate of ASD in this sample (14%), according to DSM-5 criteria applied after gold standard diagnostic instruments were given to all study participants, is lower than reported in earlier studies [7, 14], but still represents a six-fold increase above the baseline rate of ASD (2.38%) in males [33]. Discrepancies between previous community diagnoses and current research-based diagnoses are illustrative of the growing literature regarding variability in diagnostic stability in ASD (see [36] for a review) and may be attributed to a variety of factors, including change in diagnostic nosology from DSM-IV-TR to DSM-5 (e.g., lack of total overlap between previous diagnosis of PDD-NOS and current ASD diagnosis); clinical instability of diagnoses based on type of previous diagnosis, clinical setting, and, diagnosis process [37]. A larger XYY ASD cohort is required to achieve adequate statistical power for formal comparison of the broader neurodevelopmental profile of ASD within XYY, but here we did not see a unique profile in comparison to idiopathic ASD. An important goal for future work will be determining whether the observed rate of ASD in XYY is significantly elevated beyond that seen in other neurogenetic disorders with comparable levels of general developmental difficulties.

Measures of ASD symptoms, especially parent reports and/or screeners, are vulnerable to the confounding effects of impaired cognitive ability, high rates of problem behavior, and/or level of suspicion of ASD [34–36]. Indeed, in this study, we found that the sensitivity of all ASD assessment instruments was excellent, but the specificity of the dimensional questionnaire-based measures of ASD—unlike interviewer-interpreted measurement with the ADI-R and ADOS-2—was especially affected by internalizing and externalizing problems. This reflects a general psychometric challenge in ASD assessment, rather than XYY-specific phenomena [37]. Still, the limitations of these instruments in populations with high rates of problem behaviors, like XYY, must be recognized and mitigated with thorough clinical assessment and judgment. These clinical distinctions are paramount, given that they confer specific treatment priorities, which may necessitate the need for a multidisciplinary clinic type evaluation that can disentangle how these multiple symptom presentations impact functioning.

Finally, our integrative analysis suggested three separable phenotypic modules, which may provide more refined cognitive/behavioral targets for future genetic and neurobiological studies. The agnostic nature of these analyses allows for potential discovery of feature cluster that derive from unexpected co-segregations or dissociations of conventional symptom domains. For example, the “yellow” cluster in Fig. 3 combines developmental milestones with measures of adaptive behavior, while it splits features of internalizing disorders away from symptoms of externalizing psychopathology. Graph-theoretical treatment of clinical features also facilitates future integration with graph-theoretical analyses of neuroimaging data—potentially allowing for detection of linked modules of altered brain and behavior in XYY syndrome.

### Limitations and future directions

Although we quantitatively measured a wide variety of neurodevelopmental features, with a specific focus on ASD symptoms and diagnosis, the range of behaviors that can be assessed in a single study are limited by practical considerations (e.g., time constraints), primary use of caregiver questionnaires vs. inclusion of school-based measures, and availability of validated instruments. Nevertheless, access to a greater diversity of scales in future studies—including different assessment methods (e.g., caregiver vs. teacher vs. performance based) for the same developmental domain—would help to further characterize neurodevelopmental features of XYY syndrome, and more firmly resolve dissociable subsets among these features.

While our comparison of profiles with a similarly characterized ASD cohort provided some context, differential ascertainment methods between these groups may limit comparability. Relatedly, the wide age range and cross-sectional data limit our understanding of how time itself impacts the course and severity of symptom profiles described. It was also not practical to have the evaluators be masked to all information regarding previously established genetic diagnoses throughout their assessments, but this would have been ideal. We attempted to address the ascertainment bias that exists in many XYY studies by exploring differences in timing of XYY diagnosis, which may not have completely resolved the possibility of bias. However, definitive control of such biases will require new, large-scale genetic testing of population-based birth cohorts. In the meantime, studies of infant and toddler development among prenatally diagnosed groups with sex chromosome aneuploidy will be transformative.

Given the frequency of XYY and the limited (albeit substantial) cohort evaluated, this study may offer some cautiously provided guidance regarding the types of assessments and intervention that may be useful to support and improve outcomes for individuals identified with XYY, although none of these proposals should be considered unique to XYY. Based on the variability in impairments observed in this sample, it is recommended that evaluations for this population include components assessing cognitive, adaptive, and academic skills along with evaluation of behavioral problems to provide a comprehensive assessment of potentially impacted domains. Additionally, if screening indicates a referral for an ASD assessment, the use of a team evaluation comprised of autism experts using gold standard measures of ASD symptoms is recommended to increase the likelihood of the provision of accurate and stable diagnoses. Early intervention along with the later academic interventions and the potential need for targeted support in adaptive skill development should be considered.

An important, yet challenging goal for future research will be specifying the genomic mechanisms through which carriage of a supernumerary Y-chromosome can influence human neurodevelopment. These mechanisms may involve altered expression of dosage-sensitive Y-linked gametologs such as NLGN4Y [38] that are expressed in the brain [39] and have been argued to influence neurodevelopmental traits [16]. However, there are currently no means of conducting definitive tests for such hypotheses given ethical and practical obstacles to controlled experimental manipulation of brain gene expression in humans. Finally, we noted considerable variability in the phenotypic presentation of XYY, and future investigation should also seek genetic and environmental factors that can account for these inter-individual differences in expressivity among males carrying an extra Y-chromosome.

## Conclusion

Males with XYY have variable neurodevelopmental presentation, but on average, have lower cognitive, adaptive, language, and academic skills than the general population. Though still elevated relative to the general population, the rate of ASD in XYY may be lower than suggested by earlier studies. However, there is some evidence for variable ascertainment bias effects across different facets of the neurodevelopmental phenotype in XYY that can only be addressed in population-based birth cohort studies.

## Abbreviations

ADI-R: Autism Diagnostic Interview, Revised
ADOS-2: Autism Diagnostic Observation Schedule, second edition
ASD: Autism spectrum disorder
CBCL: Child Behavior Checklist
FSIQ: Full Scale Intelligence Quotient
IQ: Intelligence quotient
NVIQ: Nonverbal Intelligence Quotient
RBS-R: Repetitive Behavior Scale, Revised
SCQ: Social Communication Questionnaire
SRS-2: Social Responsiveness Scale, second edition
SSC: Simons Simplex Collection
VABS-II: Vineland Adaptive Behavior Scale, second edition
VIQ: Verbal Intelligence Quotient
WJ-IV: Woodcock-Johnson IV
